# Use of a molecular syndromic panel for the etiological diagnosis of bloodstream infections in critically ill patients: impact on clinical outcomes and antimicrobial use from a multicenter, prospective study

**DOI:** 10.1080/07853890.2026.2750724

**Published:** 2026-11-01

**Authors:** Daniele Roberto Giacobbe, Claudia Bartalucci, Greta Cattardico, Vincenzo Di Pilato, Marco Muccio, Maurizio Fedriga, Alessandro Limongelli, Alessio Signori, Alessandra Bandera, Bruno Cacopardo, Edoardo Campanella, Alessandro Caroli, Annamaria Cattelan, Marco Piscaglia, Andrea Cortegiani, Laura Curci, Gennaro De Pascale, Giuseppe Vittorio De Socio, Filippo Del Puente, Chiara Fanelli, Teresa Fasciana, Nicholas Geremia, Maddalena Giannella, Giacomo Grasselli, Chiara Maci, Ivana Maida, Davide Mangioni, Andrea Marino, Maria Mazzitelli, Maria Chiara Meloni, Marco Merli, Elena Momesso, Chiara Oltolini, Carlo Pallotto, Sandro Panese, Matteo Passerini, Emanuele Pontali, Daniele Riccucci, Matteo Rinaldi, Marco Ripa, Vincenzo Scaglione, Francesco Saverio Serino, Vincenzo Spagnuolo, Giulia Spurio, Stefania Tigano, Carlo Torti, Giovanna Travi, Laura Magnasco, Federica Portunato, Federica Briano, Malgorzata Mikulska, Lorenzo Ball, Chiara Robba, Nicolò Patroniti, Denise Battaglini, Mauro Giacomini, Gian Maria Rossolini, Maurizio Sanguinetti, Paola Morici, Anna Marchese, Antonio Vena, Matteo Bassetti

**Affiliations:** ^a^ Department of Health Sciences (DISSAL), University of Genoa, Genoa, Italy; ^b^ Clinica Malattie Infettive, AOM - IRCCS Ospedale Policlinico San Martino, Genoa, Italy; ^c^ Department of Surgical Sciences and Integrated Diagnostics (DISC), University of Genoa, Genoa, Italy; ^d^ Section of Biostatistics, Department of Health Sciences (DISSAL), University of Genoa, Genoa, Italy; ^e^ Department of Pathophysiology and Transplantation, University of Milan, Milan, Italy; ^f^ Infectious Diseases Unit, Fondazione IRCCS Ca’ Granda Ospedale Maggiore Policlinico, Milan, Italy; ^g^ Department of Clinical and Experimental Medicine, University of Catania, Catania, Italy; ^h^ Dipartimento di Scienze Biotecnologiche di Base, Cliniche Intensivologiche e Perioperatorie, Università Cattolica del Sacro Cuore, Rome, Italy; ^i^ Dipartimento di Scienze dell’Emergenza, Anestesiologiche e della Rianimazione, Fondazione Policlinico Universitario A. Gemelli IRCCS, Rome, Italy; ^j^ Infectious and Tropical Diseases Unit, Padua University Hospital, Padua, Italy; ^k^ Perioperative, Acute, Critical Care and Emergency Medicine Section, Department of Medicine, University of Cambridge, Cambridge CB2 0AW, UK; ^l^ Department of Precision Medicine in Medical, Surgical and Critical Care Area (Me.Pre.C.C.), University of Palermo, Palermo, Italy; ^m^ Department of Anaesthesia, Intensive Care and Emergency Policlinico Paolo Giaccone, Palermo, Italy; ^n^ Department of Medicine, Infectious Diseases Clinic, Santa Maria della Misericordia Hospital, University of Perugia, Perugia, Italy; ^o^ Department of Infectious Diseases, Galliera Hospital, Genoa, Italy; ^p^ Department of Medicine, Surgery, and Pharmacy, University of Sassari, Sassari, Italy; ^q^ Unit of Infectious Diseases, Department of Clinical Medicine, Ospedale “dell’Angelo”, Venice, Italy; ^r^ Unit of Infectious Diseases, Department of Clinical Medicine, Ospedale Civile “S.S. Giovanni e Paolo”, Venice; ^s^ Department of Medical and Surgical Sciences, Alma Mater Studiorum University of Bologna, Bologna, Italy; ^t^ Infectious Disease Unit, Department for Integrated Risk Management, IRCCS Azienda Ospedaliero-Universitaria di Bologna, Bologna, Italy; ^u^ Department of Anaesthesiology, Critical Care and Emergency, Fondazione IRCCS Ca’ Granda Ospedale Maggiore Policlinico, Milan, Italy; ^v^ Vita-Salute San Raffaele University, Milan, Italy; ^w^ SC Malattie Infettive, ASST Grande Ospedale Metropolitano Niguarda, Milan, Italy; ^x^ Azienda ULSS4 Veneto Orientale, UOC Anestesia e Rianimazione, Ospedale di San Donà di Piave, Piave, Italy; ^y^ Department of Infectious Disease, Luigi Sacco Hospital, ASST-FBF-Sacco, Milan, Italy; ^z^ Department of Pathophysiology and Transplantation, University of Milano, Milan, Italy; ^aa^ Unit of Infectious Diseases, IRCCS San Raffaele Scientific Institute, Milan, Italy; ^ab^ Azienda ULSS4 Veneto Orientale, UOS Malattie Infettive, UOC Medicina, Portogruaro, Italy; ^ac^ Dipartimento di Scienze Mediche e Chirurgiche, UOC Malattie Infettive, Fondazione Policlinico Universitario Agostino Gemelli IRCCS, Rome, Italy; ^ad^ Dipartimento di Sicurezza e Bioetica, Università Cattolica del Sacro Cuore, Rome, Italy; ^ae^ Anesthesia and Intensive Care, AOM - IRCCS Ospedale Policlinico San Martino, Genoa, Italy; ^af^ Department of Informatics, Bioengineering, Robotics and System Engineering (DIBRIS), University of Genoa, Genoa, Italy; ^ag^ Department of Experimental and Clinical Medicine, University of Florence, Florence, Italy; ^ah^ Microbiology and Virology Unit, Florence Careggi University Hospital, Florence, Italy; ^ai^ Dipartimento di Scienze di Laboratorio ed Ematologiche, Fondazione Policlinico Universitario A. Gemelli IRCCS, Rome, Italy; ^aj^ UO Microbiologia, AOM - IRCCS Ospedale Policlinico San Martino, Genoa, Italy; ^ak^ Department of Health Promotion, Maternal Childhood, Internal Medicine of Excellence G. D’Alessandro, University of Palermo, Palermo, Italy

**Keywords:** Rapid molecular tests, antimicrobial resistance, antimicrobial stewardship, rapid diagnosis, bloodstream infections

## Abstract

**Background:**

Bloodstream infections (BSI) frequently occur in critically ill patients and remain associated with high mortality rates.

**Methods:**

A prospective, observational, multicenter investigation was carried out through the MULTI-SITA platform to evaluate how the addition of a molecular syndromic panel, the BIOFIRE^®^ Blood Culture Identification 2 (BCID2) Panel (BCID2 panel), to conventional blood culture diagnostics influenced key clinical endpoints and antimicrobial use. The primary endpoint was all-cause mortality within 30 days following the onset of BSI.

**Results:**

Among the 126 included critically ill patients with BSI, multivariable analysis identified SOFA score (hazard ratio [HR] 1.21, 95% confidence interval [CI] 1.09–1.34, *p* < 0.001), other concomitant infections requiring antibiotic therapy (HR 2.61, 95% CI 1.18–5.79, *p* = 0.018), and BSI caused by *Acinetobacter* spp. (HR 4.35, 95% CI 1.10–17.18, *p* = 0.036), *Staphylococcus aureus* (HR 3.38, 95% CI 1.26–9.08, *p* = 0.016), or *Stenotrophomonas maltophilia* (HR 15.97, 95% CI 2.43–104.54, *p* = 0.004) as associated with increased mortality, while no association was observed between use of the BCID2 panel and mortality (HR 0.97, 95% CI 0.42–2.25, *p* = 0.941). In secondary analyses, the use of the BCID2 panel resulted in more events of either early antimicrobial de-escalation or initiation of appropriate therapy at day one from BSI onset compared with a standard blood culture workflow (55.6% vs. 31.7%, *p* = 0.015).

**Conclusion:**

In critically ill patients with BSI, implementation of a molecular syndromic panel influenced antimicrobial management decisions without affecting mortality. Additional investigations are warranted to clarify the long-term antimicrobial stewardship implications of treatment strategies guided by molecular syndromic panels-based diagnostic workflows.

## Introduction

Bloodstream infections (BSI) due to bacteria or yeasts, mainly *Candida* spp., frequently occur in critically ill patients and remain associated with high mortality rates, particularly when septic shock develops [1,2].

Achieving a rapid etiological diagnosis is essential to optimize empirical broad-spectrum antimicrobial therapy through timely de-escalation to targeted regimens, thereby supporting antimicrobial stewardship efforts aimed at reducing inappropriate antimicrobial exposure [3,4]. Furthermore, early identification of causative pathogens and resistance determinants may help clinicians promptly escalate treatment when the empirical regimen does not adequately cover unexpected microorganisms or resistance profiles.

The BIOFIRE^®^ Blood Culture Identification 2 (BCID2) Panel (BCID2 panel) is a molecular syndromic panel enabling the detection of 33 bacterial and yeast pathogens together with 10 clinically-relevant resistance genes from positive blood cultures, with a turnaround time of approximately one hour. This rapid diagnostic tool may therefore provide an earlier basis for therapeutic revision, including escalation, de-escalation, or initiation of targeted therapy in clinically stable patients.

The current prospective multicenter observational study was designed to investigate the real-life impact of the BCID2 panel use on major clinical outcomes and antimicrobial prescribing in critically ill patients with BSI.

## Material and methods

### Study setting and objectives

MULTI-SITA is a research platform established by the Italian Society of Anti-Infective Therapy (SITA) to support observational investigations on invasive bacterial and fungal infections. Within this framework, RAPID-SITA PHENOTYPES was designed as a prospective, observational, multicenter study involving Italian hospitals. The study evaluated the effect of rapid diagnostic pathways incorporating BIOFIRE^®^ panels on clinical outcomes among consecutive critically ill adult patients admitted to intensive care units (ICU) with bloodstream infection (BSI) and/or ventilator-associated bacterial pneumonia (VABP), as detailed in the Supplementary methods. The prospective study period of the RAPID-SITA PHENOTYPES study was from 1 January 2023 to 31 October 2024. The findings of the RAPID-SITA PHENOTYPES study involving patients with VABP, either isolated or accompanied by BSI, have been reported previously [5]. The present analysis focuses on patients with BSI in the absence of concomitant VABP and includes participants enrolled at 10 Italian centers (two fewer than those included in the VABP analysis; see Supplementary methods).

The outcome measures were conceptually aligned with those adopted in the previous VABP study, although all analyses were specifically related to BSI. The primary endpoint was 30-day mortality following BSI onset, as a time-to-event endpoint. Secondary endpoints included: (*i*) earlier antimicrobial therapy discontinuation, as a time-to-event endpoint with death treated as a competing risk (see statistical analysis); (*ii*) time from BSI onset to appropriate antimicrobial therapy, in days; (*iii*) a composite endpoint of antimicrobial de-escalation among patients receiving appropriate therapy from BSI onset (day 0) and initiation of appropriate therapy on day 1 after BSI onset; and (*iv*) the incidence rate of *Clostridioides difficile* infection (CDI) during follow-up.

Consistent with the previous VABP analysis [5], the composite endpoint (*iii*) was intended to capture clinical decisions potentially influenced by the availability of BCID2 panel results. This included de-escalation among patients who received appropriate empirical treatment from infection onset, which could be facilitated by the earlier availability of BCID2 panel results compared with conventional culture results. The endpoint also included initiation of appropriate therapy on day 1 in patients who did not receive appropriate treatment from day 0 (encompassing both rapid initiation and rapid escalation of therapy potentially prompted by earlier molecular diagnostic information relative to pathogen identification and phenotypic antibiogram by culture). Exclusion criteria were (*i*) age less than 18 years; (*ii*) already included in the study for a previous BSI episode. Differences in endpoints and collected variables between the analyses on VABP and BSI are available in the Supplementary methods.

### Microbiological procedures

Identification of bacterial isolates from blood culture specimens was performed by means of matrix-assisted laser desorption ionization time of flight mass spectrometry (MALDI Biotyper, Bruker Daltonics, Billerica, MA, USA; or Vitek MS MALDI-TOF mass spectrometry, bioMérieux, Craponne, France) or biochemical profiling using automated systems, depending on standard local procedures. Routine antimicrobial susceptibility testing (AST) was performed by means of automated systems (MicroScan, Beckman Coulter, Brea, CA, USA; Vitek 2, bioMérieux, Craponne, France; or Phoenix, Becton Dickinson Diagnostics, Sparks, MD, USA) according to local standard procedures. AST results were interpreted according to contemporary EUCAST clinical breakpoints.

### BCID2 panel testing

The BCID2 panel testing was performed by processing 200 µL of positive blood culture broth, following the manufacturer’s instructions. Each positive and negative assay result was automatically interpreted by the BIOFIRE Software to provide qualitative results for detection of single/multiple bacterial and yeast nucleic acids (i.e. *Acinetobacter calcoaceticus-baumannii* complex, *Bacteroides fragilis*, *Enterobacter cloacae* complex, *Escherichia coli*, *Klebsiella aerogenes*, *Klebsiella oxytoca*, *Klebsiella pneumoniae* group, *Proteus* spp., *Salmonella* spp., *Serratia marcescens*, *Haemophilus influenzae*, *Neisseria meningitidis*, *Pseudomonas aeruginosa*, *Stenotrophomonas maltophilia*, *Enterococcus faecalis*, *Enterococcus faecium, Listeria monocytogenes*, *Staphylococcus aureus*, *Staphylococcus epidermidis*, *Staphylococcus lugdunensis*, *Streptococcus agalactiae*, *Streptococcus pneumoniae*, *Streptococcus pyogenes*, *Candida albicans*, *Candida auris*, *Candida glabrata*, *Candida krusei*, *Candida parapsilosis*, *Candida tropicalis*, *Cryptococcus neoformans*/*Cryptococcus gattii,* in addition to the broader *Enterobacterales*, *Staphylococcus* spp., and *Streptococcus* spp. targets), as well as select genetic determinants associated with antimicrobial resistance (i.e. *bla*_IMP_, *bla*_KPC_, *bla*_OXA-48-like_, *bla*_NDM_, *bla*_VIM_, *mcr*-1, *bla*_CTX-M_, *mec*A/C and the composite *mec*A/C plus MREJ target*, vanA/B*).

### Definitions and data collected for the study

BSI was defined in the presence of at least one positive blood culture for bacteria or yeasts. For coagulase-negative staphylococci and other common skin contaminants, at least two consecutive blood cultures (from two separate sets/sites within 48 h) positive for the same pathogen were necessary to define BSI [6]. Besides information regarding primary and secondary outcome measures, the following demographic and clinical variables were collected as they were at the time of BSI onset: age in years; sex; diabetes mellitus; chronic obstructive pulmonary disease (COPD); New York Heart Association (NYHA) score; chronic liver disease (defined histologically as liver cirrhosis or in presence of a clinical diagnosis supported by laboratory, endoscopy, and radiologic findings [7]); chronic kidney disease (defined as estimated glomerular filtration rate <60 mL/min/1.73 m^2^); chronic intermittent hemodialysis; solid neoplasm; metastatic solid neoplasm; hematological malignancy; previous hematopoietic stem cell transplantation (HSCT); previous solid organ transplantation (SOT); human immunodeficiency virus (HIV) infection; autoimmune disease; age-adjusted Charlson Comorbidity Index [8]; previous antibiotic therapy, overall and stratified according to different agents/classes (within 6 months); previous antifungal therapy, overall and stratified according to different agents/classes (within 6 months); previous chemotherapy (within 6 months); previous steroid therapy (within 6 months); previous therapy with other immunosuppressants (within 6 months); previous major surgery (within 3 months); previous isolation of carbapenemase-producing Enterobacterales (CPE); previous isolation of carbapenem-resistant *Acinetobacter baumannii* (CRAB); previous isolation of carbapenem-resistant *Pseudomonas aeruginosa* (CRPA); previous isolation of methicillin-resistant *Staphylococcus aureus* (MRSA); previous isolation of vancomycin-resistant enterococci (VRE); days from ICU admission to BSI onset; sequential organ failure assessment (SOFA) score [9]; presence of central venous catheter (CVC); presence of septic shock [10]; presence of at least mild acute respiratory distress syndrome (ARDS) [11]; neutropenia (defined as absolute neutrophil count <500 cells/mm^3^); continuous renal replacement therapy (CRRT); extracorporeal membrane oxygenation (ECMO); presence of at least stage 1 of acute kidney injury (AKI) according to the Kidney Disease: Improving Global Outcome (KDIGO) criteria [12]; other concomitant infections requiring antibiotic therapy; etiological agents of BSI according to culture results (or BCID2 panel results in case of positive blood culture with subsequent lack of pathogen identification); days from BSI onset to appropriate antimicrobial therapy (defined as therapy with at least one agent displaying *in vitro* activity against the given BSI isolate/s; in the possible case of positive blood culture with subsequent lack of pathogen identification, appropriate therapy was defined as therapy with at least one agent considered as presumably active based on the type of microorganism/s and presence/lack of resistance genes identified by the BCID2 panel; appropriateness of antimicrobial therapy based solely on positive BCID2 panel results was assessed by two independent investigators, with cases of disagreement being resolved by a third investigator); use of cytokine blood filter/s; administration of intravenous immunoglobulins. De-escalation was defined as a reduction in the spectrum of administered antimicrobials through the discontinuation of antimicrobials or switching to an agent with a narrower spectrum [13].

### Statistical analysis

Demographic and clinical characteristics of the study population were compared, for descriptive purposes, between critically ill patients with BSI in whom the BCID2 panel was performed on positive blood cultures and critically ill patients with BSI in whom only blood cultures were processed by the conventional workflow, using the Fisher exact test or the chi-squared test for categorical variables, as appropriate, and the Wilcoxon test for continuous variables. The crude 30-day mortality from the onset of BSI was summarized graphically by means of the Kaplan-Meier method, and compared between critically ill patients with BSI in whom the BCID2 panel was performed and critically ill patients with BSI in whom only blood culture was performed using the log rank test. The primary study analysis was to assess the independent impact of the performance of the BCID2 panel on 30-day mortality. In this regard, we first performed Rubin’s multiple imputation [14]. The association of performance of the BCID2 panel and other demographics and clinical variables with 30-day mortality was first tested in univariable Cox regression models, with ICU discharge within 30 days from BSI onset as a right censoring event. Then, all the variables potentially associated with mortality in univariable comparisons (*p* < 0.10) were included in an initial multivariable Cox regression model, with subsequent selection for inclusion in the final multivariable Cox regression by means of a backward stepwise procedure. In line with the purpose of the study, the variable “BCID2 panel performed” was included in this multivariable model independent of its selection by the stepwise procedure. Variables included in the final multivariable model were also included in an additional multivariable Cox regression model also including center as shared frailty [15]. A secondary study analysis, conducted using the imputed datasets, was to assess the independent impact of the performance of the BCID2 panel on anticipating discontinuation of antimicrobial therapy (i.e. reducing length of BSI treatment). The association of performance of the BCID2 panel and other demographics and clinical variables with earlier discontinuation of antimicrobial therapy was first tested in univariable Fine-Gray models, with ICU discharge within 30 days from antimicrobial therapy initiation for BSI as a right-censoring event, and death as a competing risk [16]. Then, all the variables potentially associated with mortality in univariable comparisons (*p* < 0.10) were included in an initial multivariable Fine-Gray model, with subsequent selection for inclusion in the final multivariable Fine-Gray model by means of a backward stepwise procedure. In line with the purpose of the study, the variable “BCID2 panel performed” was included in this multivariable model independent of its selection by the stepwise procedure. Variables included in the final multivariable model were also included in an additional Fine-Gray competing risks model, accounting for center-level clustering through robust variance estimation. Of note, in Fine-Gray models for antimicrobial therapy discontinuation, a subdistribution hazard ratio (sHR) > 1.00 indicated a direction of effect towards reduction of the length of antimicrobial therapy. Other secondary analyses were aimed to compare the following descriptive outcomes between patients in whom the BCID2 panel was performed and patients in whom only blood culture was performed: (i) time to appropriate antimicrobial therapy from BSI onset, in days (compared using the Wilcoxon test); (ii) composite outcome of antimicrobial de-escalation in patients receiving appropriate therapy from infection onset or starting of an appropriate therapy from day 1 (compared using the Fisher exact test); (iii) incidence rate of CDI per 1000 patient days in ICU (compared using Poisson regression). The analyses were conducted using SAS software (version 9.4, SAS Institute Inc., Cary, NC, USA) and R Statistical Software (version 4.2.2, R Foundation for Statistical Computing, Vienna, Austria).

## Results

During the study period, 126 critically ill patients with BSI were included in the analysis. Among them, 38 patients (30.2%) underwent BCID2 panel testing in addition to conventional blood cultures workflow, whereas 88 patients (69.8%) were managed based on the results of conventional blood cultures alone (Figure 1).

**Figure 1.**
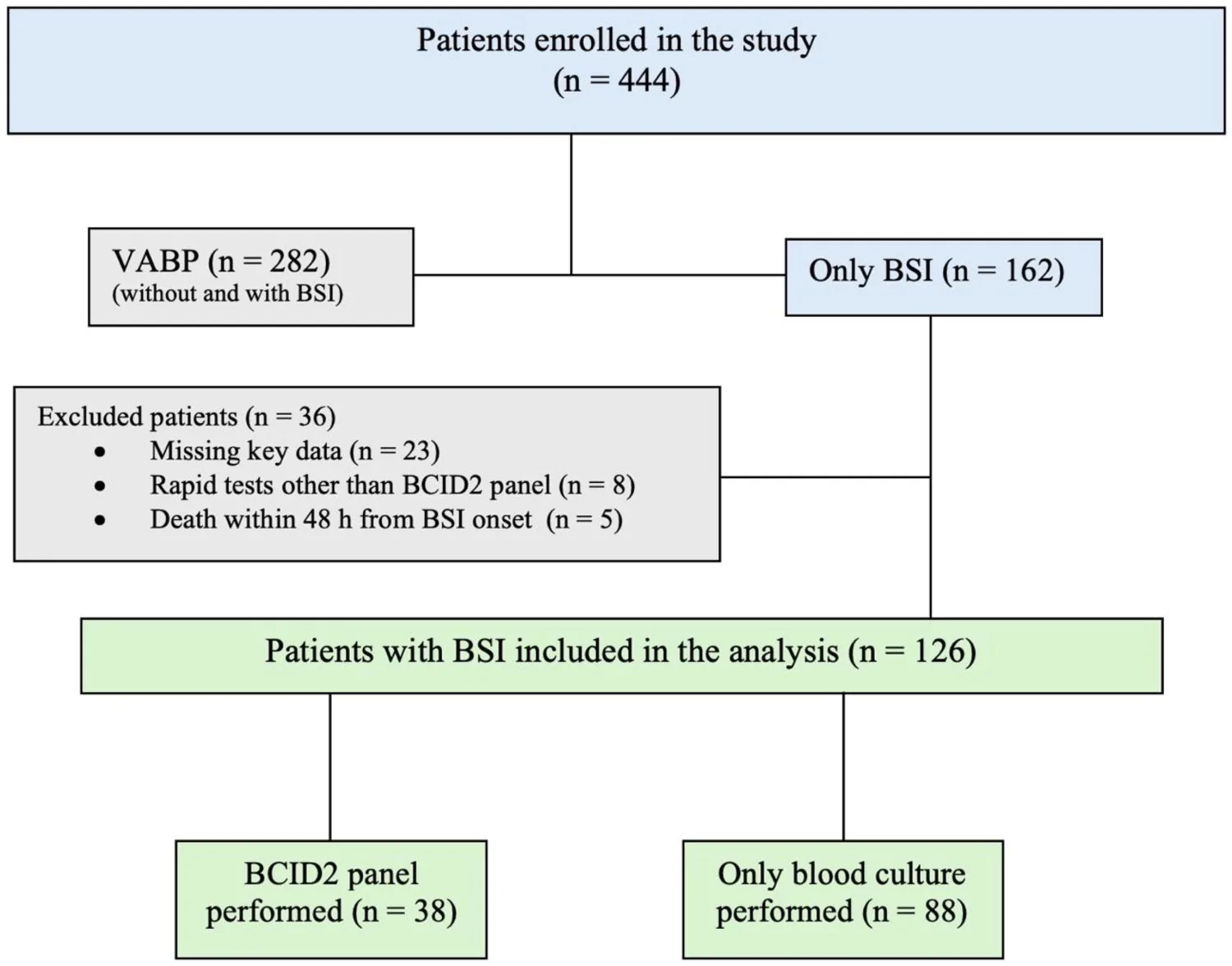
Flow-chart of the patient inclusion process. BSI, bloodstream infection; VABP, ventilator-associated bacterial pneumonia.

Baseline demographic and clinical characteristics of the study population, both overall and according to BCID2 panel testing, are summarized in Table 1. The most frequently identified, non-mutually exclusive, etiological agents of BSI were Enterobacterales (50/126, 39.7%), followed by *Enterococcus* spp. (20/126, 15.9%) and *Staphylococcus aureus* (13/126, 10.3%). Among patients who underwent BCID2 panel testing, positivity of both BCID2 panel and blood culture was observed in 94.7% of cases (36/38), whereas positivity of blood culture alone occurred in 5.3% (2/38). In patients with positivity of both diagnostic methods, the same pathogen identified by blood culture was also detected by the BCID2 panel in 31 out of 36 cases (86.1%). In 3/36 cases (8.8%), all three involving polymicrobial BSIs caused by two pathogens, the BCID2 panel identified only one of the microorganisms subsequently detected by blood culture; the missed pathogens included *Streptococcus anginosus* in two cases and *Stenotrophomonas maltophilia* in one case. In the remaining 2/36 cases (5.6%), positive blood cultures yielded growth without final pathogen identification, whereas the BCID2 panel detected *Acinetobacter calcoaceticus-baumannii* complex and *Listeria monocytogenes*, respectively. Among the two patients (5.3%) with positive blood cultures and negative BCID2 panel testing, both infections were monomicrobial and caused by *E. faecalis* and *Enterobacter* spp., respectively.

**Table 1.** Demographic and clinical characteristics.

Variables*	Total *N* = 126	BCID2 panel performed *n* = 38	Only blood culture performed *n* = 88	*p*
	No. of patients (%)	No. of patients (%)	No. of patients (%)	
**Demographics**				
Age in years, median (IQR)	65 (57–74)	62 (49–70)	66 (59–74)	0.057
Male sex	91 (72.2)	28 (73.7)	63 (71.6)	0.810
**Comorbidities and medical history**				
Diabetes mellitus	35/125 (28.0)	9 (23.7)	26/87 (29.9)	0.478
COPD	16/124 (12.9)	1 (2.6)	15/86 (17.4)	0.022
NYHA score, median (IQR)	*n* = 117 1 (1–2)	*n* = 36 1 (1–2)	*n* = 81 1 (1–2)	0.910
Chronic liver disease	11 (8.7)	4 (10.5)	7 (8.0)	0.733
Chronic kidney disease	31/125 (24.8)	12 (31.6)	19/87 (21.8)	0.246
Chronic intermittent haemodialysis	13/125 (10.4)	7 (18.4)	6/87 (6.9)	0.063
Solid neoplasm	25/125 (20)	6 (15.8)	19/87 (21.8)	0.437
Metastatic solid neoplasm	2/79 (2.5)	1/25 (4.0)	1/54 (1.9)	0.536
Hematological malignancy	10/125 (8.0)	4 (10.5)	6/87 (6.9)	0.490
Previous HSCT	3/125 (2.4)	1 (2.6)	2/87 (2.3)	1.000
Previous SOT	6/125 (4.8)	4 (10.5)	2/87 (2.3)	0.069
HIV infection	3/122 (2.5)	1 (2.6)	2/84 (2.4)	1.000
Autoimmune disease	8/124 (6.5)	2 (5.3)	6/86 (7.0)	1.000
Age-adjusted Charlson Comorbidity Index, median (IQR)	4 (3–6)	4 (3–6)	4 (3–6)	0.524
Previous antibiotic therapy	43 (34.1)	7 (18.4)	36 (40.9)	0.015
Previous aminoglycosides	1 (0.8)	0 (0.0)	1 (1.1)	1.000
Previous fluoroquinolones	6 (4.8)	2 (5.3)	4 (4.5)	1.000
Previous macrolides	2 (1.6)	0 (0.0)	2 (2.3)	1.000
Previous trimethoprim/sulfamethoxazole	1 (0.8)	0 (0.0)	1 (1.1)	1.000
Previous metronidazole/clindamycin	6 (4.8)	0 (0.0)	6 (6.8)	0.177
Previous antistaphylococcal penicillins	1 (0.8)	0 (0.0)	1 (1.1)	1.000
Previous semisynthetic aminopenicillins	5 (4.0)	0 (0.0)	5 (5.7)	0.321
Previous piperacillin/tazobactam	24 (19.0)	3 (7.9)	21 (23.9)	0.036
Previous cefazolin	3 (2.4)	0 (0.0)	3 (3.4)	0.553
Previous ceftriaxone/cefotaxime	7 (5.6)	1 (2.6)	6 (6.8)	0.674
Previous ceftazidime/cefepime	2 (1.6)	0 (0.0)	2 (2.3)	1.000
Previous ceftaroline/ceftobiprole	7 (5.6)	1 (2.6)	6 (6.8)	0.674
Previous ceftolozane/tazobactam	7 (5.6)	2 (5.3)	5 (5.7)	1.000
Previous ceftazidime/avibactam	1 (0.8)	0 (0.0)	1 (1.1)	1.000
Previous cefiderocol	1 (0.8)	0 (0.0)	1 (1.1)	1.000
Previous carbapenems	15 (11.9)	1 (2.6)	14 (15.9)	0.038
Previous imipenem/relebactam	0 (0.0)	0 (0.0)	0 (0.0)	–
Previous meropenem/vaborbactam	0 (0.0)	0 (0.0)	0 (0.0)	–
Previous fosfomycin	1 (0.8)	0 (0.0)	1 (1.1)	1.000
Previous polymyxins	0 (0.0)	0 (0.0)	0 (0.0)	–
Previous tigecycline	6 (4.8)	0 (0.0)	6 (6.8)	0.177
Previous glycopeptides	9 (7.1)	2 (5.3)	7 (8.0)	0.722
Previous daptomycin	9 (7.1)	1 (2.6)	8 (9.1)	0.276
Previous linezolid	13 (10.3)	1 (2.6)	12 (13.6)	0.107
Previous antifungal therapy	16/125 (12.8)	2 (5.3)	14/87 (16.1)	0.145
Previous azoles	4/125 (3.2)	0 (0.0)	4/87 (4.6)	0.313
Previous echinocandins	13/125 (10.4)	2 (5.3)	11/87 (12.6)	0.341
Previous polyenes	2/125 (1.6)	0 (0.0)	2/87 (2.3)	1.000
Previous chemotherapy	8/124 (6.5)	3 (7.9)	5/86 (5.8)	0.700
Previous steroid therapy	13/123 (10.6)	3 (7.9)	10/85 (11.8)	0.753
Previous therapy with immunosuppressant	10/120 (8.3)	3/37 (8.1)	7/83 (8.4)	1.000
Previous major surgery	58/121 (47.9)	16/36 (44.4)	42/85 (49.4)	0.617
Previous isolation of CPE	1/125 (0.8)	0 (0.0)	1/87 (1.1)	1.000
Previous KPC-producing CPE	0/125 (0.0)	0 (0.0)	0/87 (0.0)	–
Previous MBL-producing CPE	1/125 (0.8)	0 (0.0)	1/87 (1.1)	1.000
Previous OXA-48-producing CPE	0/125 (0.0)	0 (0.0)	0/87 (0.0)	–
Previous isolation of CRAB	1 (0.8)	0 (0.0)	1 (1.1)	1.000
Previous isolation of CRPA	3 (2.4)	1 (2.6)	2 (2.3)	1.000
Previous isolation of MRSA	3 (2.4)	0 (0.0)	3 (3.4)	0.553
Previous isolation of VRE	4 (3.2)	1 (2.6)	3 (3.4)	1.000
**Variables at BSI onset**				
Days from ICU admission to BSI onset, median (IQR)	*n* = 125 2 (0–12)	0 (0–8)	*n* = 87 4 (0–15)	0.044
SOFA Score, median (IQR)	*n* = 124 7 (4–10)	9 (6–12)	*n* = 86 6.5 (4–10)	0.079
Presence of CVC	119 (94.4)	33 (86.8)	86 (97.7)	0.026
Presence of septic shock	61 (48.4)	23 (60.5)	38 (43.2)	0.074
Presence of ARDS	53 (42.1)	13 (34.2)	40 (45.5)	0.241
Neutropenia	9/125 (7.2)	6 (15.8)	3/87 (3.4)	0.023
CRRT	29 (23.0)	11 (28.9)	18 (20.5)	0.299
ECMO	3 (2.4)	0 (0.0)	3 (3.4)	0.553
KDIGO stage of AKI, median (IQR)	*n* = 125 1 (0–2)	1 (0–3)	*n* = 87 0 (0–1)	0.079
Other concomitant infections requiring antibiotic therapy	36 (28.6)	11 (28.9)	25 (28.4)	0.951
Causative agents and BSI therapy**				
*Pseudomonas aeruginosa*	5 (4.0)	2 (5.3)	3 (3.4)	0.637
*Acinetobacter* spp.	8 (6.3)	5 (13.2)	3 (3.4)	0.053
Members of Enterobacterales	50 (39.7)	18 (47.4)	32 (36.4)	0.247
*Staphylococcus aureus*	13 (10.3)	3 (7.9)	10 (11.4)	0.753
*Streptococcus pneumoniae*	2 (1.6)	1 (2.6)	1 (1.1)	0.514
*Haemophilus influenzae*	0 (0.0)	0 (0.0)	0 (0.0)	–
*Stenotrophomonas maltophilia*	2 (1.6)	1 (2.6)	1 (1.1)	0.514
*Enterococcus* spp.	20 (15.9)	3 (7.9)	17 (19.3)	0.107
*Candida* spp.	8 (6.3)	0 (0.0)	8 (9.1)	0.104
Days from BSI onset to appropriate antimicrobial therapy, median (IQR)	*n* = 115 1 (0–2)	*n* = 36 1 (0–1.2)	*n* = 79 1 (0–2)	0.771
Cytokine blood filtration	3/125 (2.4)	2 (5.3)	1/87 (1.1)	0.219
Intravenous immunoglobulins				
Polyclonal intravenous immunoglobulins	2/125 (1.6)	0 (0.0)	2/87 (2.3)	1.000
IgM-enriched intravenous immunoglobulins	0/125 (0.0)	0 (0.0)	0/87 (0.0)	–

Denominators reported in presence of missing values. AKI, acute kidney injury; ARDS, acute respiratory distress syndrome; BCID2, blood culture identification 2; BSI, bloodstream infection; COPD, chronic obstructive pulmonary disease; CPE, carbapenemase-producing Enterobacterales; CRAB, carbapenem-resistant Acinetobacter baumannii; CRPA, carbapenem-resistant Pseudomonas aeruginosa; CRRT, continuous renal replacement therapy; CVC, central venous catheter; ECMO, extracorporeal membrane oxygenation; HIV, human immunodeficiency virus; HSCT, hematopoietic stem cell transplantation; ICU, intensive care unit; IgM, immunoglobulin M; IQR, interquartile range; KDIGO, Kidney Disease: Improving Global Outcomes; KPC, Klebsiella pneumoniae carbapenemase; MBL, metallo β-lactamases; NYHA, New York Heart Association; OXA, oxacillinase; SOFA, sequential organ failure assessment; SOT, solid organ transplantation.

* Results are presented as No. of patients/Total of patients unless otherwise indicated. ** Non-mutually exclusive.

Resistance genes were identified by BCID2 panel testing in 31.6% of tested patients (12/38), with the following non-mutually exclusive distribution: CTX-M (5/12, 41.7%), *mec*A/C (4/12, 33.3%), KPC (4/12, 33.3%), and OXA-48-like (1/12, 8.3%). Concordance between phenotypic resistance patterns and resistance genes detected by the BCID2 panel was observed in 10 of 12 cases (83.3%). Two apparently discordant cases were identified: one patient with *Enterobacter* spp. bloodstream infection carrying an OXA-48-like determinant but remaining susceptible to carbapenems, and one patient with *Streptococcus* spp. bloodstream infection in whom *mec*A/C and CTX-M genes were detected while the isolate showed phenotypic susceptibility to penicillin and other β-lactams.

As illustrated in Figure 2, unadjusted 30-day cumulative mortality was 30.5% (confidence interval [CI] 15.6%-46.9%) among patients undergoing BCID2 panel testing and 26.5% (CI 16.7%-37.4%) among those managed with blood culture alone (log-rank test, *p* = 0.470). Univariable and multivariable analyses evaluating factors associated with 30-day mortality are presented in Supplementary Table S1 and Table 2, respectively. In the final multivariable model (model A), higher SOFA score (HR 1.21, 95% CI 1.09–1.34, *p* < 0.001), other concomitant infections requiring antibiotic therapy (HR 2.61, 95% CI 1.18–5.79, *p* = 0.018), *Acinetobacter* spp. as causative agent of BSI (HR 4.35, 95% CI 1.10–17.18, *p* = 0.036), *Staphylococcus aureus* as causative agent of BSI (HR 3.38, 95% CI 1.26–9.08, *p* = 0.016), and *Stenotrophomonas maltophilia* as causative agent of BSI (HR 15.97, 95% CI 2.43–104.54, *p* = 0.004) were independently associated with increased mortality. Conversely, no significant association between BCID2 panel testing and mortality was detected (HR 0.97, 95% CI 0.42–2.25, *p* = 0.941). Similar effect directions were observed in model B, which additionally accounted for center as shared frailty, although associations involving other concomitant infections requiring antibiotic therapy and *Acinetobacter* spp. as causative agent of BSI no longer reached statistical significance (Table 2).

**Figure 2.**
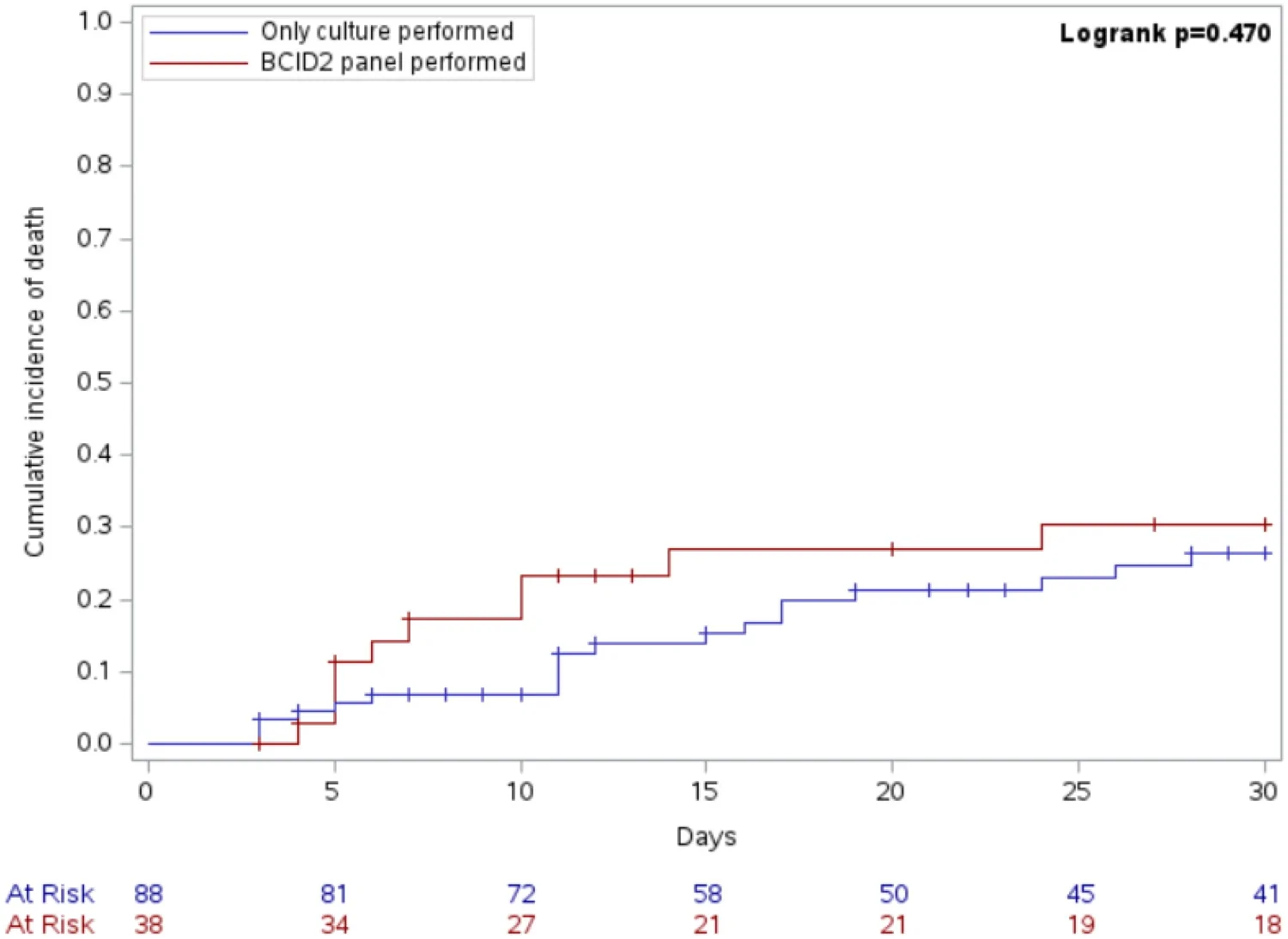
Unadjusted cumulative mortality up to Day 30 in patients with BSI. Unadjusted cumulative mortality up to Day 30 in patients with BSI in whom the BCID2 panel was performed (red line) and patients with BSI in whom only blood culture was performed (blue line). The time of origin was set as the day of BSI onset. Death was the event of interest and right-censoring was applied at the end of follow-up (ICU discharge or Day 30, whichever came first). BCID2, blood culture identification 2; BSI, bloodstream infection; ICU, intensive care unit.

**Table 2.** Multivariable analysis of factors associated with 30-day mortality.

	Multivariable backward (Model A)		Shared frailty (Model B)	
Variable	HR (95% CI)	p-value	HR (95% CI)	p-value
BCID2 panel performed	0.97 (0.42–2.25)	0.941	1.02 (0.43–2.46)	0.959
SOFA score	1.21 (1.09–1.34)	<0.001	1.18 (1.07–1.31)	0.001
Other concomitant infections requiring antibiotic therapy	2.61 (1.18–5.79)	0.018	1.80 (0.71–4.56)	0.218
*Acinetobacter* spp. as causative agent of BSI	4.35 (1.10–17.18)	0.036	2.89 (0.76–10.96)	0.119
*Staphylococcus aureus* as causative agent of BSI	3.38 (1.26–9.08)	0.016	4.03 (1.41–11.50)	0.009
*Stenotrophomonas maltophilia* as causative agent of BSI	15.97 (2.43–104.54)	0.004	24.78 (3.92–156.56)	<0.001

Only variables retained in the final multivariable model after backward stepwise selection are presented. Variables included in the initial multivariable model were the following: BCID2 panel performed; Solid neoplasm; Age-adjusted Charlson Comorbidity Index; Previous antibiotic therapy; Previous piperacillin/tazobactam; SOFA score; Presence of septic shock; KDIGO stage of AKI; Other concomitant infections requiring antibiotic therapy; *Acinetobacter* spp. as causative agent of BSI; *Staphylococcus aureus* as causative agent of BSI; *Stenotrophomonas maltophilia* as causative agent of BSI. The variable BCID2 panel performed was retained in the final multivariable model independent of stepwise backward selection, in line with the purpose of the study. BCID2, blood culture identification 2; CI, confidence interval; HR, hazard ratio; SOFA, sequential organ failure assessment.

Regarding secondary analyses evaluating factors associated with timing of antimicrobial discontinuation, results of univariable and multivariable analyses are reported in Supplementary Table S2 and Table 3, respectively. In the final multivariable model (model C), septic shock was associated with delayed antimicrobial discontinuation (sHR 0.53, 95% CI 0.31–0.91, *p* = 0.020). In contrast, autoimmune disease (sHR 2.42, 95% CI 1.02–5.74, *p* = 0.045), prior treatment with semisynthetic aminopenicillins (sHR 2.36, 95% CI 1.18–4.72, *p* = 0.016), previous ceftriaxone/cefotaxime therapy (sHR 2.54, 95% CI 1.09–5.93, *p* = 0.032), ECMO support (sHR 3.32, 95% CI 1.03–10.69, *p* = 0.044), and *Streptococcus pneumoniae* as etiological agent of BSI (sHR 3.70, 95% CI 2.27–6.02, *p* < 0.001) were independently associated with earlier discontinuation. No significant association was observed for BCID2 panel testing (sHR 0.68, 95% CI 0.37–1.26, *p* = 0.222). Comparable directions of effect estimates were confirmed in model D, which additionally adjusted for center-level clustering using robust variance estimation, although the association of autoimmune disease with earlier discontinuation was no longer statistically significant (Table 3).

**Table 3.** Multivariable analysis of factors associated with earlier antimicrobial discontinuation.

	Multivariable backward (Model C)		Cluster-adjusted model (Model D)	
Variable	sHR (95% CI)	p-value	sHR (95% CI)	p-value
BCID2 panel performed	0.68 (0.37–1.26)	0.222	0.68 (0.41–1.14)	0.144
Autoimmune disease	2.42 (1.02–5.74)	0.045	2.43 (0.69–8.58)	0.166
Previous semisynthetic aminopenicillins	2.36 (1.18–4.72)	0.016	2.36 (1.01–5.50)	0.048
Previous ceftriaxone/cefotaxime	2.54 (1.09–5.93)	0.032	2.54 (1.71–3.77)	<0.001
Presence of septic shock	0.53 (0.31–0.91)	0.020	0.53 (0.42–0.68)	<0.001
ECMO	3.32 (1.03–10.69)	0.044	3.32 (1.44–7.65)	0.005
*Streptococcus pneumoniae* as causative agent of BSI	3.70 (2.27–6.02)	<0.001	3.70 (2.94–4.67)	<0.001

Fine-Gray model with discontinuation of antimicrobial therapy as the event of interest and death as competing risk. Only variables retained in the final multivariable model after backward stepwise selection are presented. Variables included in the initial multivariable model were the following: BCID2 panel performed; Autoimmune disease; Previous macrolides; Previous semisynthetic aminopenicillins; Previous ceftriaxone/cefotaxime; Previous ceftazidime/cefepime; Previous azoles; SOFA score; Presence of septic shock; ECMO; KDIGO stage of AKI; *Streptococcus pneumoniae* as causative agent of BSI. The variable BCID2 panel performed was retained in the final multivariable model independent of stepwise backward selection, in line with the purpose of the study. BCID2, blood culture identification 2; CI, confidence interval; ECMO, extracorporeal membrane oxygenation; sHR, subdistribution hazard ratio.

Median time to appropriate antimicrobial therapy was 1 day (interquartile range [IQR] 0–1) in patients undergoing BCID2 panel testing and 1 day (IQR 0–2) in those managed with blood culture alone (*p* = 0.771). Nevertheless, the composite endpoint including antimicrobial de-escalation among patients already receiving appropriate therapy from day 0 and initiation of appropriate therapy on day 1 among those not appropriately treated at day 0 occurred more frequently in patients who underwent BCID2 panel testing compared with those managed with conventional blood cultures alone (55.6% [20/36] vs. 31.7% [25/79], *p* = 0.015). The individual components of the composite endpoint are graphically summarized in Figure 3. Only one episode of CDI was documented, occurring in a patient undergoing BCID2 panel testing. Consequently, Poisson regression analysis was not performed.

**Figure 3.**
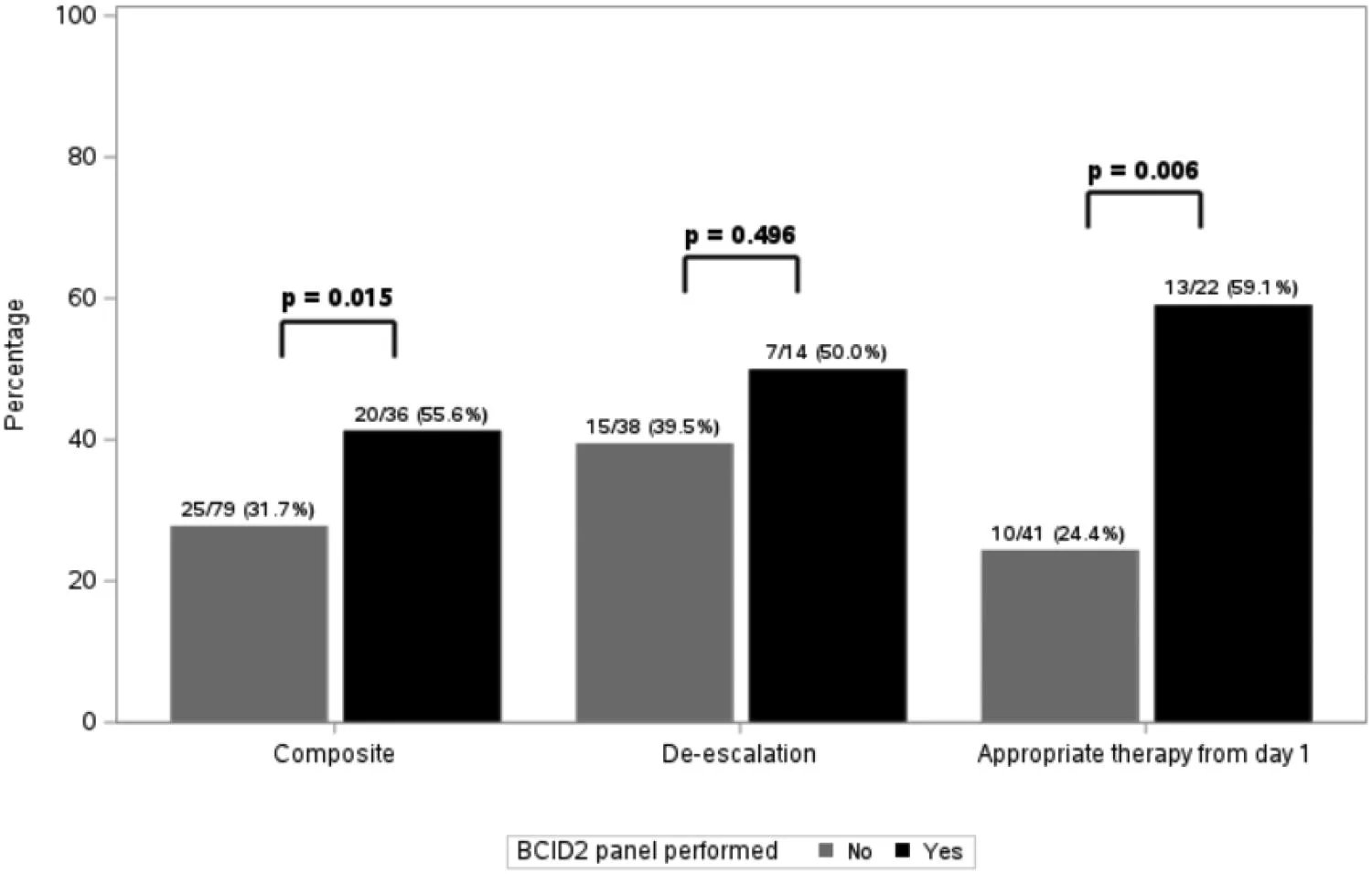
Composite outcome of antibiotic de-escalation in patients who received appropriate therapy from BSI onset (day 0) and initiation of an appropriate therapy from day 1 in those who did not initiate an appropriate therapy on day 0. p values from chi square test. BSI, bloodstream infection. In patients with BCID2 panel performed, de-escalation was obtained through discontinuation of anti-Gram positive agents (5/7, 71.4%), or change to an agent with narrower spectrum (2/7, 28.6%). In patients with only culture performed, de-escalation was obtained through discontinuation of anti-Gram positive agents (3/15, 20.0%), discontinuation of anti-Gram negative agents (6/15, 40.0%), change to an agent with narrower spectrum (5/15, 33.3%), or discontinuation of ani-anaerobes agents (1/15, 6.7%). In patients with BCID2 panel performed, initiation of appropriate therapy from day 1 occurred because of initiation of any therapy on day 1 (10/13, 76.9%), or change of therapy on day 1 (3/13, 23.1%). In patients with only culture performed, initiation of appropriate therapy from day 1 occurred because of initiation of any therapy on day 1 (7/10, 70.0%), or change of therapy on day 1 (3/10, 30.0%).

## Discussion

The use of the BCID2 panel in critically ill patients with BSI did not impact 30-day mortality and time to antimicrobial therapy discontinuation. Use of rapid molecular testing was associated with different antimicrobial use compared with treatment strategies guided exclusively by conventional blood culture results.

If our results are confirmed in larger cohorts, the absence of an effect on mortality may support the safety of antimicrobial decisions guided by rapid molecular syndromic testing and aligns both with antimicrobial stewardship objectives (e.g. earlier initiation of targeted therapy) and with our previous findings in patients with VABP [5]. The effect of the BCID2 panel implementation on mortality among critically ill patients with BSI has been explored in prior investigations. A retrospective single-center study including 181 ICU patients with BSI receiving inappropriate empirical therapy, among whom 33 underwent BCID2 panel testing, identified BCID2-guided therapeutic adjustment as an independent predictor of reduced 28-day mortality [17]. Similarly, a partly prospective study performed across three ICUs, comparing retrospective data before implementation with prospective data after BCID2 panel introduction, reported lower 30-day mortality among the 86 patients who underwent BCID2 panel testing out of 185 critically ill patients with BSI [18]. By contrast, another retrospective single-center study comparing pre- and post-implementation periods, including 70 patients in each cohort, found no significant difference in 28-day mortality [19]. Our findings are consistent with the latter study. The lack of mortality benefit may be explained by the relatively short time to appropriate antimicrobial therapy even among patients managed without BCID2 panel testing, likely reflecting the high prevalence of adequate empirical treatment in our cohort (as testified by the overall median time from BSI onset to appropriate therapy of 1 day). Nevertheless, BCID2 panel implementation appeared to promote antimicrobial use more consistent with stewardship principles, as demonstrated by the higher frequency of the composite endpoint including early de-escalation or initiation of appropriate therapy at day 1 from BSI onset in those who did not initiate appropriate therapy at day 0. Of note, while the relative low number of this latter subgroup is in line with the overall lack of effect in mortality in our entire cohort, the possibility of a favorable survival effect by expanding this subgroup in larger studies cannot be excluded.

Another factor that may have contributed to not showing improved survival in patients undergoing BCID2 panel testing is the higher infection severity of patients tested with the BCID2 panel, who showed higher rates of septic shock and higher median SOFA scores than patients managed with blood cultures alone, although the adjustment for SOFA score in the final multivariable model calls for caution in identifying this reason as the only explanation. In a similar way, the lack of effect of BCID2 panel testing on earlier antimicrobial discontinuation may also be related to the greater infection severity among patients undergoing molecular testing, since an heavier burden of disease could justify longer treatment durations. From this perspective, the absence of significantly prolonged therapy compared with less severely ill patients undergoing conventional diagnostics could still be interpreted as a favorable finding. However, as for the mortality endpoint, the partial adjustment for severity in multivariable analysis, where septic shock was retained in the final model, warrants cautious interpretation and deserves further investigation. Beyond septic shock, among additional variables independently associated with antimicrobial discontinuation (but in this case with earlier discontinuation, contrarily to septic shock) in both standard and center-adjusted multivariable models were previous exposure to amoxicillin/clavulanate, ceftriaxone, or cefotaxime, as well as S*. pneumoniae* as the causative pathogen of BSI. Although also these associations require further validation, they could indicate a greater probability of adequate empirical treatment, either because of clinician awareness of prior antibiotic exposure or because of the generally low resistance rates of *S. pneumoniae* to β-lactams in Italy [20]. However, it is worth noting that such associations with earlier discontinuation, including the additional one we found for ECMO, deserve due caution and further investigation before drawing any definite interpretation, in view of the reduced frequency of these variables in our cohort.

Several limitations of the present study should be acknowledged, many of which overlap with those reported in our previous VABP analysis [5]. First, despite the numerically similar mortality reported in patients who underwent BCID2 panel testing vs. those who did not, our main analysis should be regarded as exploratory due to its reduced power, thereby requiring validation in larger cohorts. Second, clinicians’ responses to BCID2 panel results were not standardized across participating centers, potentially introducing heterogeneity in therapeutic decision-making. To mitigate this issue, shared frailty models and center-level clustering adjustments were applied. Third, the observational real-world design may have introduced selection bias, since clinicians may have considered BCID2 panel testing unnecessary in some patients managed exclusively with conventional cultures, as suggested by the higher prevalence of septic shock in BCID2 panel group. Fourth, the lack of information on prognosis after ICU discharge was addressed using right-censoring, that nonetheless may not always be completely non-informative in this specific setting. Fifth, the clinical utility of BCID2 panel testing may vary according to the microbiological epidemiology and prevalence of multidrug-resistant organisms in different settings. For example, centers with higher endemicity of carbapenemase-producing pathogens could potentially derive greater benefit from rapid molecular diagnostics, particularly regarding time to appropriate therapy. Sixth, although our primary and secondary endpoints were specifically selected to assess survival and immediate stewardship-related outcomes, the study was not designed to evaluate the long-term impact of BCID2-guided therapeutic decisions. Dedicated investigations will therefore be needed to determine whether rapid molecular syndromic testing confers sustained stewardship advantages over time, especially considering that current evidence increasingly supports the absence of detrimental effects on major short-term clinical outcomes such as mortality. Seventh, despite rapid communication of the BCID2 panel results through institutional stewardship and diagnostic pathways at all participating centers, occasional delays in result notification cannot be excluded, particularly during night shifts or periods with limited staffing, representing a potential unmeasured confounders. Finally, the composite secondary endpoint combining antimicrobial de-escalation in patients already receiving appropriate therapy at BSI onset and initiation of appropriate therapy by day 1, although previously proposed in our VABP analysis to capture clinical decisions occurring before identification and susceptibility testing through blood cultures (and therefore maximize the detectable impact of rapid molecular testing), still requires external validation.

In conclusion, our exploratory findings indicate that BCID2 panel testing in critically ill patients with BSI may influence antimicrobial management decisions without affecting mortality. Further studies are warranted to confirm our results in larger cohorts and to clarify the long-term antimicrobial stewardship implications of therapeutic strategies guided by BCID2 panel testing.

## Supplementary Material

supplementary material_revised.docx

## Data Availability

The data presented in this study will be available from the corresponding author on reasonable request and provided all regulatory and privacy requirements are fulfilled.
