# Supplementary material for: Use of a molecular syndromic panel for the etiological diagnosis of bloodstream infections in critically ill patients: impact on clinical outcomes and antimicrobial use from a multicenter, prospective study
Source: Ann Med. 2026 Nov 1;58(1):2750724. doi: 10.1080/07853890.2026.2750724 (PMC13729619; doi:10.1080/07853890.2026.2750724)
Supplement: Supplementary file 1 [file IANN_A_2750724_SM6657.docx]

**SUPPLEMENTARY METHODS**

In the RAPID-SITA PHENOTYPES study, etiological diagnosis could be achieved by either BIOFIRE® panels (PNplus panel on bronchoalveolar lavage samples and BCID2 panel on positive blood cultures for VABP and BSI, respectively), standard microbiological cultures (BAL cultures and blood cultures for VABP and BSI, respectively), or both, performed on samples collected within 24 hours before and 24 hours after the onset of the infection. All microbiological tests were requested and performed according to local clinical practice, in accordance with the observational design.

The present analysis focuses on patients with BSI in the absence of concomitant VABP. The following differences should be acknowledged compared with our previous analysis of VABP patients [1]:

- Overall, 9/12 centers included in the VABP analysis provided data also for the BSI analysis. One center provided data only for the BSI analysis.
- The variable previous VRE colonization was not among variables included in the VABP analysis, while it was included in the present analysis of BSI. For completeness of information, no previous VRE colonization was reported in patients included in the VABP analysis.
- The endpoint incidence rate of candidemia per 1000 patient days in ICU was not included in the present analysis of BSI as *Candida* spp. could also be causative agents of the index BSI (with recurrent infections, if present, potentially biasing results). Although not included due to the above bias, it was calculated for transparency. The incidence rate of candidemia was 1.13 per 1000 patient-days in patients who underwent BCID2 panel testing, while it was 0.66 per 1000 patient-days in patients who did not. The incidence rate ratio was 1.71 (95% CI 0.15-18.81, p = 0.663), indicating a non-statistically significant difference. This estimate should be interpreted with caution due to the very small number of candidemia events observed (n = 3).
- Separated, slightly different multiple imputation procedures were used in the two analyses (one on the VABP cohort and one on the BSI cohort), in order to not have a leakage of cohort-specific characteristics biasing imputation. For transparency, imputed data is available from the corresponding author together with baseline data to allow for full reproducibility, on reasonable request and provided all regulatory and privacy requirements are fulfilled.

**Abbreviations.** BCID2, blood culture identification 2; BSI, bloodstream infection; CI, confidence interval; ICU, intensive care unit; VABP; ventilator-associated bacterial pneumonia; VRE, vancomycin-resistant enterococci.

**References**

[1] Giacobbe DR, Cattardico G, Bartalucci C, et al. Use of a molecular syndromic panel for the etiological diagnosis of ventilator-associated bacterial pneumonia: impact on clinical outcomes and antibiotic use from a multicenter, prospective study. Crit Care. 2025;29(1):403.

**Supplementary Table S1. Univariable analysis of factors associated with 30-day mortality**

| **Variables*** | **Univariable analysis** | |
| --- | --- | --- |
|  | **HR (95% CI)** | **p-value** |
| BCID2 panel performed | 1.33 (0.62-2.85) | 0.471 |
| Age in years | 1.02 (0.99-1.05) | 0.242 |
| Male sex | 0.84 (0.38-1.84) | 0.656 |
| Diabetes mellitus | 1.65 (0.77-3.56) | 0.201 |
| COPD | 0.84 (0.29-0.75) | 0.746 |
| NYHA score | 1.33 (0.85-2.08) | 0.214 |
| Chronic liver disease | 1.15 (0.35-3.81) | 0.816 |
| Chronic kidney disease | 1.30 (0.57-2.95) | 0.529 |
| Chronic intermittent haemodialysis | 1.45 (0.50-4.21) | 0.491 |
| Solid neoplasm | 2.15 (0.98-4.74) | 0.056 |
| Metastatic solid neoplasm | 3.38 (0.51-22.23) | 0.206 |
| Hematological malignancy | 0.37 (0.05-2.71) | 0.327 |
| Previous HSCT | 1.38 (0.19-10.18) | 0.749 |
| Previous SOT* | - | - |
| HIV infection* | - | - |
| Autoimmune disease | 0.57 (0.08-4.25) | 0.588 |
| Age-adjusted Charlson Comorbidity Index | 1.11 (0.99-1.26) | 0.085 |
| Previous antibiotic Therapy | 0.29 (0.10-0.81) | 0.020 |
| Previous aminoglycosides* | - | - |
| Previous fluoroquinolones* | - | - |
| Previous macrolides | 1.82 (0.25-13.42) | 0.555 |
| Previous trimethoprim/sulfamethoxazole* | - | - |
| Previous metronidazole/clindamycin | 0.99 (0.13-7.36) | 0.993 |
| Previous antistaphylococcal penicillins* | - | - |
| Previous semisynthetic aminopenicillins* | - | - |
| Previous piperacillin/tazobactam | 0.15 (0.02-1.10) | 0.062 |
| Previous cefazolin | 1.50 (0.20-11.01) | 0.692 |
| Previous ceftriaxone/cefotaxime | 0.69 (0.09-5.07) | 0.712 |
| Previous ceftazidime/cefepime* | - | - |
| Previous ceftaroline/ceftobiprole | 0.44 (0.06-3.27) | 0.425 |
| Previous ceftolozane/tazobactam | 0.44 (0.06-3.27) | 0.425 |
| Previous ceftazidime/avibactam* | - | - |
| Previous cefiderocol* | - | - |
| Previous carbapenems | 0.61 (0.15-2.56) | 0.498 |
| Previous imipenem/relebactam* | - | - |
| Previous meropenem/vaborbactam* | - | - |
| Previous fosfomycin* | - | - |
| Previous polymyxins* | - | - |
| Previous tigecycline* | - | - |
| Previous glycopeptides | 1.11 (0.26-4.68) | 0.885 |
| Previous daptomycin | 0.36 (0.05-2.61) | 0.310 |
| Previous linezolid | 0.31 (0.04-2.27) | 0.248 |
| Previous antifungal therapy | 0.20 (0.03-1.46) | 0.112 |
| Previous azoles | 0.98 (0.13-7.45) | 0.985 |
| Previous echinocandins | 0.25 (0.03-1.86) | 0.177 |
| Previous polyenes* | - | - |
| Previous chemotherapy | 0.49 (0.07-3.61) | 0.486 |
| Previous steroid therapy | 0.30 (0.04-2.20) | 0.236 |
| Previous therapy with immunosuppressant | 0.40 (0.05-2.93) | 0.366 |
| Previous major surgery | 1.28 (0.60-2.71) | 0.518 |
| Previous isolation of CPE* | - | - |
| Previous KPC-producing CPE* | - | - |
| Previous MBL-producing CPE* | - | - |
| Previous OXA-48-producing CPE* | - | - |
| Previous isolation of CRAB* | - | - |
| Previous isolation of CRPA* | - | - |
| Previous isolation of MRSA* | - | - |
| Previous isolation of VRE | 1.12 (0.15-8.22) | 0.913 |
| Days from ICU admission to BSI onset | 0.98 (0.95-1.01) | 0.244 |
| SOFA Score | 1.20 (1.11-1.32) | <0.001 |
| Presence of CVC* | - | - |
| Presence of septic shock | 2.16 (1.01-4.65) | 0.049 |
| Presence of ARDS | 0.93 (0.45-1.95) | 0.856 |
| Neutropenia | 2.17 (0.75-6.23) | 0.151 |
| CRRT | 0.75 (0.31-1.85) | 0.538 |
| ECMO* | - | - |
| KDIGO stage of AKI | 1.55 (1.15-2.08) | 0.004 |
| Other concomitant infections requiring antimicrobial therapy | 3.15 (1.52-6.53) | 0.002 |
| *Pseudomonas aeruginosa* as causative agent of BSI* | - | - |
| *Acinetobacter* spp. as causative agent of BSI | 2.82 (0.98-8.11) | 0.054 |
| Members of Enterobacterales as causative agent of BSI | 0.97 (0.46-2.06) | 0.944 |
| *Staphylococcus aureus* as causative agent of BSI | 2.16 (0.88-5.31) | 0.094 |
| *Streptococcus pneumoniae* as causative agent of BSI* | - | - |
| *Haemophilus influenzae* as causative agent of BSI* | - | - |
| *Stenotrophomonas maltophilia* as causative agent of BSI | 33.93 (6.82-168.59) | <0.001 |
| *Enterococcus* spp. as causative agent of BSI | 1.38 (0.56-3.38) | 0.486 |
| *Candida* spp. as causative agent of BSI* | - | - |
| Days from BSI onset to appropriate antimicrobial therapy** | 1.01 (0.79-1.28) | 0.961 |
| Cytokine blood filtration* | - | - |
| Polyclonal intravenous immunoglobulins* | - | - |
| IgM-enriched intravenous immunoglobulins* | - | - |

**Supplementary Table S1 legend.** Analyses done only for variables with a sufficient number of events. AKI, acute kidney injury; ARDS, acute respiratory distress syndrome; BCID2, blood culture identification 2; BSI, bloodstream infection; CI confidence interval; COPD, chronic obstructive pulmonary disease; CPE, carbapenemase-producing Enterobacterales; CRAB, carbapenem-resistant Acinetobacter baumannii; CRPA, carbapenem-resistant Pseudomonas aeruginosa; CRRT, continuous renal replacement therapy; CVC, central venous catheter; ECMO, extracorporeal membrane oxygenation; HIV, human immunodeficiency virus; HR, hazard ratio; HSCT, hematopoietic stem cell transplantation; ICU, intensive care unit; IgM, immunoglobulin M; KDIGO, Kidney Disease: Improving Global Outcomes; KPC, Klebsiella pneumoniae carbapenemase; MBL, metallo β-lactamases; NYHA, New York Heart Association; OXA, oxacillinase; SOFA, sequential organ failure assessment; SOT, solid organ transplantation
* Analysis not performed due to lack of events in a category. ** Modelled as time dependent.

**Supplementary Table S2. Univariable analysis of factors associated with earlier antimicrobial discontinuation**

| **Variables*** | **Univariable analysis** | |
| --- | --- | --- |
|  | **sHR (95% CI)** | **p-value** |
| BCID2 panel performed | 0.53 (0.30-0.93) | 0.028 |
| Age in years | 1.00 (0.99-1.02) | 0.649 |
| Male sex | 1.02 (0.60-1.75) | 0.936 |
| Diabetes mellitus | 0.78 (0.44-1.39) | 0.399 |
| COPD | 1.36 (0.72-2.58) | 0.349 |
| NYHA score | 1.05 (0.79-1.41) | 0.736 |
| Chronic liver disease | 0.73 (0.29-1.83) | 0.502 |
| Chronic kidney disease | 0.78 (0.45-1.36) | 0.387 |
| Chronic intermittent haemodialysis | 0.78 (0.40-1.51) | 0.460 |
| Solid neoplasm | 1.00 (0.54-1.86) | 0.992 |
| Metastatic solid neoplasm* | - | - |
| Hematological malignancy | 0.62 (0.25-1.55) | 0.311 |
| Previous HSCT | 1.32 (0.35-4.93) | 0.679 |
| Previous SOT | 0.80 (0.14-4.69) | 0.805 |
| HIV infection | 1.54 (0.51-4.66) | 0.444 |
| Autoimmune disease | 2.65 (1.32-5.33) | 0.006 |
| Age-adjusted Charlson Comorbidity Index | 0.94 (0.86-1.02) | 0.127 |
| Previous antibiotic Therapy | 1.26 (0.79-2.00) | 0.336 |
| Previous aminoglycosides* | - | - |
| Previous fluoroquinolones | 0.43 (0.11-1.71) | 0.229 |
| Previous macrolides | 4.46 (2.88-6.91) | <0.001 |
| Previous trimethoprim/sulfamethoxazole* | - | - |
| Previous metronidazole/clindamycin | 1.12 (0.37-3.33) | 0.845 |
| Previous antistaphylococcal penicillins* | - | - |
| Previous semisynthetic aminopenicillins | 2.21 (1.02-4.79) | 0.045 |
| Previous piperacillin/tazobactam | 1.27 (0.77-2.08) | 0.347 |
| Previous cefazolin | 1.14 (0.38-3.45) | 0.816 |
| Previous ceftriaxone/cefotaxime | 2.51 (1.08-5.84) | 0.032 |
| Previous ceftazidime/cefepime | 1.30 (1.00-1.70) | 0.051 |
| Previous ceftaroline/ceftobiprole | 1.10 (0.54-2.24) | 0.803 |
| Previous ceftolozane/tazobactam | 1.17 (0.54-2.53) | 0.685 |
| Previous ceftazidime/avibactam* | - | - |
| Previous cefiderocol* | - | - |
| Previous carbapenems | 1.21 (0.60-2.44) | 0.601 |
| Previous imipenem/relebactam* | - | - |
| Previous meropenem/vaborbactam* | - | - |
| Previous fosfomycin* | - | - |
| Previous polymyxins* | - | - |
| Previous tigecycline | 1.35 (0.63-2.93) | 0.444 |
| Previous glycopeptides | 1.09 (0.47-2.54) | 0.842 |
| Previous daptomycin | 0.95 (0.43-2.10) | 0.891 |
| Previous linezolid | 1.52 (0.87-2.67) | 0.142 |
| Previous antifungal therapy | 1.33 (0.74-2.42) | 0.343 |
| Previous azoles | 2.37 (1.28-4.39) | 0.007 |
| Previous echinocandins | 1.15 (0.56-2.34) | 0.704 |
| Previous polyenes | 2.43 (0.58-10.15) | 0.222 |
| Previous chemotherapy | 0.32 (0.07-1.48) | 0.145 |
| Previous steroid therapy | 1.69 (0.81-3.52) | 0.163 |
| Previous therapy with immunosuppressant | 0.70 (0.25-1.94) | 0.496 |
| Previous major surgery | 0.76 (0.47-1.24) | 0.270 |
| Previous isolation of CPE* | - | - |
| Previous KPC-producing CPE* | - | - |
| Previous MBL-producing CPE* | - | - |
| Previous OXA-48-producing CPE* | - | - |
| Previous isolation of CRAB* | - | - |
| Previous isolation of CRPA | 0.86 (0.35-2.14) | 0.753 |
| Previous isolation of MRSA | 1.07 (0.38-2.98) | 0.903 |
| Previous isolation of VRE | 0.34 (0.06-1.87) | 0.213 |
| Days from ICU admission to BSI onset | 1.00 (0.99-1.01) | 0.695 |
| SOFA Score | 0.92 (0.86-0.99) | 0.020 |
| Presence of CVC | 1.36 (0.42-4.37) | 0.605 |
| Presence of septic shock | 0.50 (0.30-0.82) | 0.006 |
| Presence of ARDS | 1.31 (0.82-2.10) | 0.260 |
| Neutropenia | 1.01 (0.51-2.02) | 0.974 |
| CRRT | 1.07 (0.64-1.78) | 0.791 |
| ECMO | 4.01 (1.28-12.51) | 0.017 |
| KDIGO stage of AKI | 0.78 (0.62-0.98) | 0.037 |
| Other concomitant infections requiring antibiotic therapy | 0.80 (0.46-1.40) | 0.436 |
| *Pseudomonas aeruginosa* as causative agent of BSI | 2.05 (0.84-5.00) | 0.115 |
| *Acinetobacter* spp. as causative agent of BSI | 0.48 (0.17-1.34) | 0.161 |
| Members of Enterobacterales as causative agent of BSI | 1.04 (0.63-1.69) | 0.890 |
| *Staphylococcus aureus* as causative agent of BSI | 0.50 (0.22-1.16) | 0.106 |
| *Streptococcus pneumoniae* as causative agent of BSI | 1.93 (1.39-2.69) | <0.001 |
| *Haemophilus influenzae* as causative agent of BSI* | - | - |
| *Stenotrophomonas maltophilia* as causative agent of BSI | 1.24 (0.08-19.43) | 0.877 |
| *Enterococcus* spp. as causative agent of BSI | 0.88 (0.45-1.72) | 0.698 |
| *Candida* spp. as causative agent of BSI | 0.69 (0.27-1.79) | 0.445 |
| Days from BSI onset to appropriate antimicrobial therapy** | 1.03 (0.89-1.19) | 0.719 |
| Cytokine blood filtration | 1.40 (0.86-2.28) | 0.174 |
| Polyclonal intravenous immunoglobulins | 1.89 (0.65-5.49) | 0.242 |
| IgM-enriched intravenous immunoglobulins* | - | - |

**Supplementary Table S2 legend.** Analyses done only for variables with a sufficient number of events. AKI, acute kidney injury; ARDS, acute respiratory distress syndrome; BCID2, blood culture identification 2; BSI, bloodstream infection; CI confidence interval; COPD, chronic obstructive pulmonary disease; CPE, carbapenemase-producing Enterobacterales; CRAB, carbapenem-resistant Acinetobacter baumannii; CRPA, carbapenem-resistant Pseudomonas aeruginosa; CRRT, continuous renal replacement therapy; CVC, central venous catheter; ECMO, extracorporeal membrane oxygenation; HIV, human immunodeficiency virus; HSCT, hematopoietic stem cell transplantation; ICU, intensive care unit; IgM, immunoglobulin M; KDIGO, Kidney Disease: Improving Global Outcomes; KPC, Klebsiella pneumoniae carbapenemase; MBL, metallo β-lactamases; NYHA, New York Heart Association; OXA, oxacillinase; sHR, subdistribution hazard ratio; SOFA, sequential organ failure assessment; SOT, solid organ transplantation
* Analysis not performed due to lack of events in a category. ** Modelled as fixed, despite the time of origin of the Fine-Gray analysis corresponding to the start of any therapy (either initially appropriate or inappropriate); this choice was supported by the non-significance of a time interaction term (p = 0.749).
